# Isolation and identification of *Alternaria alstroemeriae* causing postharvest black rot in citrus and its control using curcumin-loaded nanoliposomes

**DOI:** 10.3389/fmicb.2025.1555774

**Published:** 2025-03-04

**Authors:** Jie Li, Zuyun Zhang, Ping Yang, Yu Zhao, Jiaxin Fang, Tingting Yang, Ruopeng Yang

**Affiliations:** ^1^College of Biological and Agricultural Sciences, Honghe University, Mengzi, China; ^2^College of Chemistry and Resources Engineering, Honghe University, Mengzi, China; ^3^Yunnan Province International Joint Laboratory of Green Food (China-Vietnam), College of Chemistry and Resources Engineering, Honghe University, Mengzi, China

**Keywords:** curcumin-loaded nanoliposomes, postharvest diseases, pathogenicity, antifungal, morphological analysis

## Abstract

Citrus black rot caused by the pathogen *Alternaria alstroemeriae* severely affects the growth and production of citrus industry. In order to further elucidate the pathogen of citrus fruit rot in Yunnan Province, the pathogenic fungi causing citrus fruit rot were identified through isolation and purification, pathogenicity testing, morphological characteristics, and rDNA ITS sequence analysis. Meanwhile, we synthesized curcumin-loaded nanoliposomes, a potential management approach to control citrus postharvest pathogen, and conducted *vitro* and *vivo* experiment to investigate the effects of different curcumin-loaded nanoliposomes treatments inhibitory effect to pathogen *A. alstroemeriae*. The results showed that the pathogenic fungi of citrus rot diseases were *A. alstroemeriae, Rhizopus arrhizus, Aspergillus flavus* and *Penicillium digitatum.* The curcumin-loaded nanoliposomes had inhibitory effect on *A. alstroemeriae*, *in vitro* experiment showed that the minimum fungicidal concentration (MIC) of curcumin-loaded nanoliposomes against the hyphae growth of *A. alstroemeriae* was 10 μmol/L, and 4MIC treatment significantly reduced the occurrence of black rot in citrus fruit *in vivo* test. Curcumin-loaded nanoliposomes also enhanced the activities of the enzymes PPO, APX, POD, PAL, GR and CAT of citrus, decreased the O_2_^−^ production rate. The accumulation of ASA, GSH and hydrogen radical scavenging rate in *Citrus reticulata* Blanco ‘Orah’ were increased in the curcumin-loaded nanoliposomes treatment fruit, which may be directly responsible for the delayed onset of black rot disease. Furthermore, curcumin-loaded nanoliposomes treatment maintained the quality of citrus fruit by delaying the TSS, TA degradation and higher level of total phenolics and flavonoid contents in citrus fruit. Overall, our findings revealed that curcumin-loaded nanoliposomes, functioning as a plant elicitor, could effectively modulate physiological enzyme activities to confer the black rot resistance in citrus, which highlighted the potential of curcumin-loaded nanoliposomes for sustainable agricultural practices.

## 1 Introduction

There are 135 countries that cultivate and produce citrus fruits in the world, its planting area and annual yield rank first among all fruits (Noorizadeh et al., 2022). Citrus fruit production was estimated to be about 103 million tons for 2021/22 (U.S. Department of Agriculture, 2023). After citrus harvest, significant losses can occur during storage, transportation and marketing (Munera et al., 2023). The decay caused by fungal diseases are the main causes of postharvest losses in most citrus fruits (Riolo et al., 2024). Specifically, the pathogen *Penicillium digitatum, Penicillium italicum, Geotrichum citri-aurantii* can infect fruit during citrus postharvest storage (Duan et al., 2021). Citrus black rot is a postharvest disease that can lead to internal decay of citrus fruits, several *Alternaria* species can cause this disease (Güney et al., 2023). *Alternaria* black rot is one of the most important diseases of several citrus fruits worldwide (Akimitsu et al., 2003). The widespread occurrence of *Alternaria* spp. on citrus fruit might pose a serious problem for citrus industry worldwide (Garganese et al., 2016). The *Alternaria* can infect various plants or fruit, there are significant differences in the species and pathogenicity of *Alternaria* in different regions of the world (Garganese et al., 2019). Accordingly, isolating strains of *Alternaria* and investigating about the epidemiology, fungicide activity from different regions, which are crucial for reducing postharvest losses in the local citrus industry. Chemical fungicides have been widely used in the past few decades to control postharvest decay of citrus fruits (Li et al., 2024). However, the extensive use of fungicides may lead to drug-resistant fungal strains, thus there is an urgent need to explore safe and effective fungicides.

Curcumin is a natural polyphenol that can be extracted from *Curcuma longa L.* rhizomes (Gomez-Estaca et al., 2012). Research indicated that curcumin has various biological activities (Zhang et al., 2024). The curcumin has broad-spectrum antimicrobial activities, including antibacterial (Li et al., 2024), antiviral (Mathew and Hsu, 2018), and antifungal (Song et al., 2020). However, curcumin has simple degradability, lesser solubility, and poor bioactivity, (Katiyar et al., 2024). To overcome the above disadvantages, studies have shown that combining delivery carrier substances with curcumin is an effective method to improve its stability and biological activity. The carrier substances included nanoparticles (Zhong et al., 2024), emulsions (Zhan et al., 2024) and liposomal systems (Niu et al., 2012). The research revealed the particle size of the phospholipid bilayer was decreased and fluidity improved when curcumin was encapsulated by liposome (Chen et al., 2022).

In this study, we hypothesized that curcumin-loaded nanoliposomes have potent antifungal activity against citrus postharvest pathogen *A. alstroemeriae*, which would induce the postharvest decay of citrus fruit caused by this pathogen. This study aimed to evaluate the efficacy of curcumin-loaded nanoliposomes in controlling postharvest citrus black rot. The specific objectives were (1) to isolate and identify the causal agent, (2) to evaluate the effect of curcumin-loaded nanoliposomes on postharvest citrus decay caused by the causal agent, and (3) to identify the possible mechanism by which curcumin-loaded nanoliposomes inhibits this disease.

## 2 Materials and methods

### 2.1 Curcumin-loaded nanoliposomes

The curcumin-loaded nanoliposomes was provided by Suzhou will nanobiotech Co., Ltd. (Shuzhou, China). Liposomes composition including phosphatidylcholine 1 (w/w) %, Tween-80 20 (w/w) %, Diethylene glycol ether 50 (w/w) %, Water 20 (w/w) %, Curcumin 9 (w/w) %.

### 2.2 Isolation of fungal strains from decayed citrus fruit

In the August of 2023, the mature Nanfeng tangerine was harvested in Liming Village (23°52′N latitude, 102°52′E longitude) Jianshui county of Yunnan province, China. During storage, citrus fruit displaying typical symptoms of decay were recorded. To isolate and identify the pathogens that cause fruit decay. The naturally decaying citrus fruits were selected and rinse under tap water, after that disinfected the fruit peel with 75% ethanol for 1 min, then disinfected with 0.5% sodium hypochlorite for 3 min, and finally wash three times with sterile distilled water. To excised the symptomatic areas of citrus fruits with a sterile scalpel and transfer them to potato dextrose agar plates for incubation at 25°C for 3 days. Select fungal colonies based on their morphology and streak them on potato dextrose agar medium for preliminary separation. After culturing at 25°C for 3 days, observe the colony morphology and repeat separation until a single colony was obtained. All isolates were stored in 20% glycerol at −80°C refrigerator until use.

### 2.3 Morphologic and microscopic characterization

The purified strains were cultivated on PDA plate and incubated in a 25°C incubator for 3 days. Observation of spore morphology by an OLYMPUS optical microscope (Tokyo, Japan).

### 2.4 DNA extraction, PCR amplification, and phylogenetic analysis of the pathogenic fungus

Extract the total DNA of each isolated fungus using the fungal Genomic DNA Isolation Kit (SolarBio, Beijing, China) according to the manufacturer’s instructions. PCR amplification was performed using 2 × Taq PCR Mastermix and fungal ITS universal primers ITS1 (5’-TCGTAGTGGAACCTGCGG-3′) and ITS4 (5’-TCGTAGTGGA ACCTGCGG-3′). The reaction system consists of 2xTaq 25.0 *μ*L, ITS1 4.0 μL, ITS4 4.0 μL, total DNA extraction solution 2 μL, and ddH2O 15 μ L. The PCR reaction conditions are: 94°C for 3 min; 94°C 30s, 5°C 30s, 72°C 45 s, 30 cycles; 72°C for 10 min. After reaction, the products were amplified in 1.5% agarose gel electrophoresis for specific detection, and sent to Shanghai Sangon Biological Engineering Technology and Services Co., Ltd. for sequencing (Shanghai, China). Perform sequence alignment of the sequencing results in GenBank in NCBI, and use the Neighbor Joining method in MEGA 11.0 software to perform systematic analysis and construct a phylogenetic tree.

### 2.5 Pathogenicity test

To fulfill Koch’s postulates, surface-sterilized healthy citrus fruit was selected and disinfected the surface of the fruits with 75% ethanol, rinse 2–3 times with sterile water, and thoroughly wipe the surface moisture with sterilized filter paper. The flavedo of citrus fruit were wounded with a sterilized punch, and using sterilized inoculation needles to pick a PDA plugs (5 mm in diameter) and place it on the surface of the fruit. As a control, sterile PDA agar plugs were used. Following inoculation, both treatment groups for citrus were placed in a sterilized plastic container on a layer of moist filter paper at 25°C for 8 days. The experiments were repeated twice. After four isolated fungi infected citrus fruits, samples isolated from the lesion were cultured and then compared with the original strain.

### 2.6 Curcumin-loaded nanoliposomes antifungal activity against pathogen *in vitro*

#### 2.6.1 Determination of the minimum inhibitory concentration and minimum fungicidal concentration

The minimal inhibitory concentration (MIC) and minimal fungicidal concentration (MFC) of the curcumin-loaded nanoliposomes were determined in sterile cultural dishes using the method described by Niu et al. (2022) with minor modification. For each dish, 100 μL of curcumin-loaded nanoliposomes were dispensed on PDA medium at doses between 0.01–100 μmol/L. Then, 200 μL of conidial suspension (1 × 10^6^ spores/mL) were added to each dish. Cultural dishes were incubated at 25°C for 72 h. For tested pathogen four replicas were performed. MIC was considered the lowest concentration of curcumin-loaded nanoliposomes at which each pathogen did not grow. To determine MFC, 10 μL of the concentration corresponding to the MIC and higher concentrations were sub-cultured on PDA dishes and incubated at 25°C for 72 h. Accordingly, MFC was defined as the lowest concentration which prevented any mycelium growth.

#### 2.6.2 Determination of malondialdehyde content

The impact of curcumin-loaded nanoliposomes on membrane integrity in *A. alstroemeriae* was assessed through the quantification of MDA content. The MDA content was determined based on the thiobarbituric acid reactive substrates (TBARS) assay (Zhang et al., 2022). Mycelial samples subjected to varying curcumin-loaded nanoliposomes concentrations (0, 1/2MIC, 1MIC) at different time points (0, 12, 24, and 48 h) were collected for analysis.

#### 2.6.3 Determination of alkaline phosphatase activity

*A. alstroemeriae* hyphae cultured for 48 h was collected and treated with curcumin-loaded nanoliposomes (0, 1/2MIC and 1MIC) for 0, 12, 24 and 48 h. The extracellular AKP activity was examined using the AKP kit (Solarbio Science and Technology Co., Ltd., Beijing, China).

### 2.7 Curcumin-loaded nanoliposomes antifungal activity against pathogen *in vivo*

The impact of curcumin-loaded nanoliposomes on black rot in *Citrus reticulata* Blanco ‘Orah’ was investigated by creating wounds (3.0 mm wide and 1.5 mm deep) in the fruit and sprayed curcumin-loaded nanoliposomes solution of 0 (control), 2MIC and 4MIC to the peel. After 6 h, 20 μL of *A. alstroemeriae* spore suspension (1 × 10^6^ spores/mL) was injected into the wounds. Incubated fruit were placed in a plastic box and place them in an incubator at 25 ± 2°C with 95% relative humidity for 15 d. Each treatment consisted of three replicates.

#### 2.7.1 Determination of disease incidence and lesion diameter

Disease incidence and lesion diameter were evaluated every 3 days interval after inoculation. Each treatment contained 48 fruits and the experiment was conducted three times.

#### 2.7.2 Effect of curcumin-loaded nanoliposomes on the citrus POD, CAT and APX activity

In an ice bath, 1 g of citrus fruit peel was ground in 1 mL extraction buffer solution and centrifuged (4°C; 12,000 × *g*; 30 min) to extract peroxidase (POD), catalase (CAT) and ascorbate peroxidase (APX), and the supernatant was collected for further measurement. POD, CAT and APX activities were assessed using Cai’s method with minor modifications (Cai et al., 2024). For the POD activity assay, 3.7 mL of reaction solutions, consisting of 0.5 mL crude enzyme solution, 3 mL guaiacol, 0.2 mL 0.5 mol L^−1^ H_2_O_2_ (diluted with 0.05 mol L^−1^ pH 6.8 PBS). The CAT activities were determined using a 3 mL reaction solution consisting of 2.9 mL 20 mmol L^−1^ H_2_O_2_, 0.1 mL crude enzyme solution. For the APX activity assay, 3 mL of reaction solutions, 3 mL of reaction solutions, consisting of 2.6 mL reaction buffer solution (contains 0.1 mmol L^−1^ EDTA,0.5 mmol L^−1^ascorbic acid), 0.1 mL crude enzyme solution and 2 mmol L^−1^ H_2_O_2_.

#### 2.7.3 Effect of curcumin-loaded nanoliposomes on the citrus PPO and PAL activity

Crude enzymes were extracted according to the previous method (Cheng et al., 2015) with some modifications. All enzyme extraction procedures were conducted at 4°C. The activities of PPO and PAL in citrus fruit were determined according to the protocols of Xu et al. (2021). For the PPO activity assay, 3 mL of reaction solutions, consisting of 2.7 mL 0.1 mol L^−1^ PBS, 0.1 mL catechol, 0.2 mL crude enzyme solution. For the PAL activity assay, 4.1 mL of reaction solutions, consisting of 3.0 mL 50 mmol L^−1^、pH 8.8 PBS, 0.5 mL 20 mmol L^−1^ L-phenylalanine, 0.5 mL crude enzyme solution.

#### 2.7.4 Effect of curcumin-loaded nanoliposomes on the citrus total phenol and flavonoid content

The determination of flavonoid and phenol contents was performed as reported by Cai et al. (2024) method with some modifications. Briefly, 0.6 g citrus pericarp powder was added to 1 mL 1% HCl-methanol solution (v/v), and centrifuged at 12,000 × *g* at 4°C for 10 min to collect the supernatant solution. The absorbance was determined at 280 nm for total phenols content, and 325 nm for phenolic contents.

#### 2.7.5 Effect of curcumin-loaded nanoliposomes on the citrus glutathione reductase activity, reduced glutathione content and O_2_^−^ production rate and hydrogen radical scavenging rate

The GR activity and GSH content in citrus fruit were determined according to the method of Zhang et al. (2024) with minor modifications. The O_2_^−^ production rate and hydrogen radical scavenging rate was measured using the slightly modified method of Zhu et al. (2021). The hydrogen radical scavenging rate was determined according to Dai et al. (2023) method.

#### 2.7.6 Effect of curcumin-loaded nanoliposomes on the citrus Total soluble solids, titratable acid and ascorbic acid contents

TSS content was determined by portable digital refractometer (PAL-13810, Japan). Titratable acidity and ascorbic acid content was determined according to Aubert et al. (2019) method.

### 2.8 Statistical analysis

Microsoft Excel 2016 and GraphPad 9.0 were used to analyze the experimental data. All data were analyzed by one-way analysis of variance using SPSS 22.0 (SPSS Inc., Chicago, United States).

## 3 Results

### 3.1 Morphological identification of isolated strains from decayed citrus fruit

Four strains were isolated from decayed citrus fruits, denoted as strains A, B, C, and D. The colony of strain A is fluffy, and the hyphae initially white and gradually turn green. The substrate color is white, the hyphae have 1–3 branches at the top, resembling a broom. There are conidiophores and conidia, and the conidia are colorless and nearly spherical (Supplementary Figures S1A, S2A). Strain B is filamentous with long hyphae, initially gray white in color, gradually filling the petri dish, and eventually turning gray brown to black. The conidia are mostly round and dark brown, with no branching or septa on the mycelium (Supplementary Figures S1B, S2B). Strain C is scattered and initially grows white hyphae, gradually producing green powdery mold. The bottom matrix is white, and microscopic examination shows the presence of a large number of spherical conidia with a rough surface (Supplementary Figures S1C, S2C). Strain D is filamentous with white hyphae and spreading outward, gradually producing dark green powdery mold. Its conidiophores are broom shaped, with 1–2 branches at the top, forming a slender spindle shape. Spores grow in tandem on the stem. Spores are oval or cylindrical (Supplementary Figures S1D, S2D).

### 3.2 Pathogenicity test of isolated strains

Citrus fruit inoculated with isolate A was rotten after 9 days. The sites of inoculation had lesions can vary from small specks to large pockmarks, indicating severe pathogenicity toward the citrus fruit (Supplementary Figure S3A). Citrus fruit inoculated with isolate B was rotten after 6 days, the citrus fruits soften with water stains, white hyphae was growing (Supplementary Figure S3B). Citrus fruit inoculated with isolate C was rotten after 6 days. Initially, white mycelium grows at the center of the equator, gradually producing blue powdery mold, and finally the fruit soften (Supplementary Figure S3C). The citrus fruit inoculated with strain D shown decayed symptoms after 5 days. Firstly, white mycelium grows and spreads outward, and then dark green powdery spreads throughout the fruit (Supplementary Figure S3D).

### 3.3 Molecular identification of the isolate strains

The ITS region of four isolates were amplified and sequenced. A phylogenetic tree of the four isolates were constructed (Supplementary Figures S4A–D). Isolate A was clustered together with strains of *A. alstroemeriae isolate* LS-PH-L-51 and exhibited 68% sequence similarity (Supplementary Figure S4A). These results strongly suggest that the isolate belongs to the species *A. alstroemeriae*.

### 3.4 *In vitro* inhibitory effects of curcumin-loaded nanoliposomes against *Alternaria alstroemeriae*

#### 3.4.1 MIC and MFC of curcumin-loaded nanoliposomes

**Figure 1:**
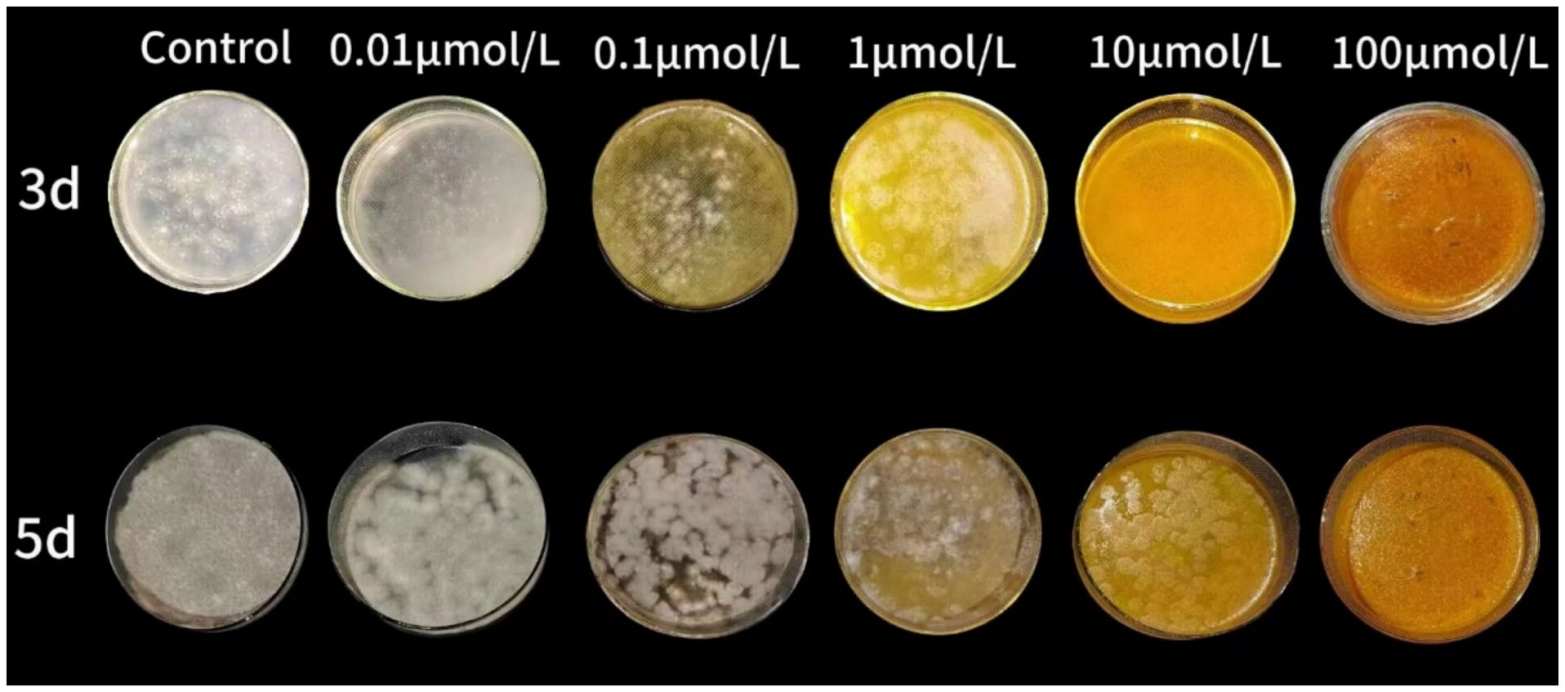
The inhibitory effect of curcumin-loaded nanoliposomes on *A. alstroemeriae* growth *in vitro.*

The curcumin-loaded nanoliposomes possessed MIC at 10 μmol/L and MFC at 100 μmol/L after 2 days and 5 days incubation, respectively. The colony diameter of nanoliposomes treated group was smaller than the control group. As the concentration of curcumin-loaded nanoliposomes increased, the inhibition rate increased. At 100 μmol/L or higher concentrations of curcumin-loaded nanoliposomes, the inhibition rate reached to 100% (Figure 1).

#### 3.4.2 Impact of curcumin-loaded nanoliposomes on MDA contents of *Alternaria alstroemeriae*

**Figure 2:**
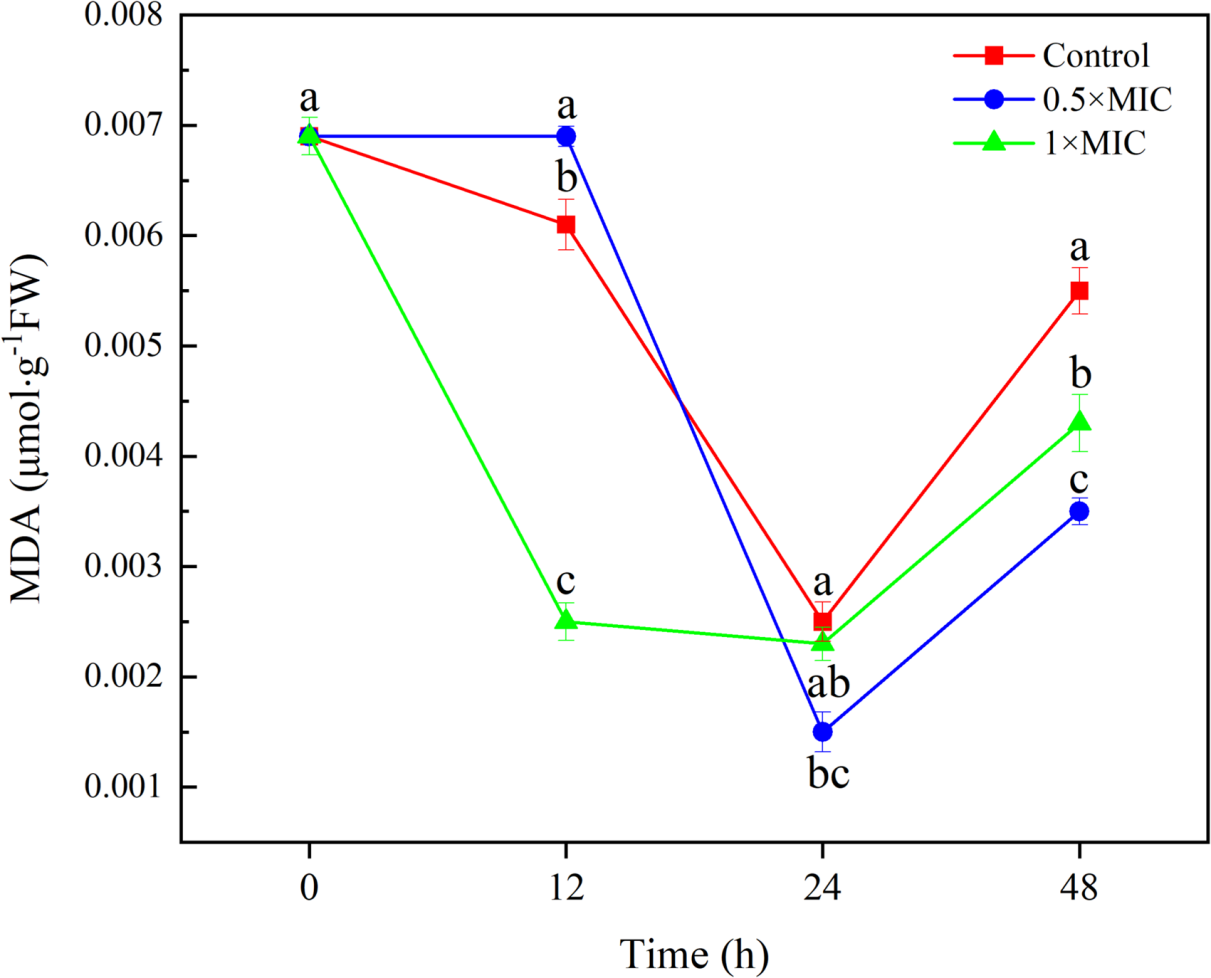
The inhibitory effect of curcumin-loaded nanoliposomes on *A. alstroemeriae* MDA contents. Values are the mean ± standard deviation (SD) of three replicates. Different superscripts letters mean significantly different (*p* < 0.05).

The MDA content of 1/2MIC treatment group was 0.014 μmol/g, which was significantly different from the control group with value 0.025 μmol/g at 24 h (*p* < 0.05). The MDA content of 1MIC treatment group was 0.023 μmol/g, and there was no significant difference compared to the control group with value 0.025 μmol/g at 48 h, the MDA content in the 0.5MIC treatment group was 0.0353 μmol/g. The MDA content of the 0.5MIC and 1MIC treatment groups were 0.0353 μmol/g and 0.0427 μmol/g at 48 h, respectively, which were significantly lower than the control group with value 0.055 μmol/g (*p* < 0.05; Figure 2).

#### 3.4.3 Impact of curcumin-loaded nanoliposomes on AKP activity of *Alternaria alstroemeriae*

**Figure 3:**
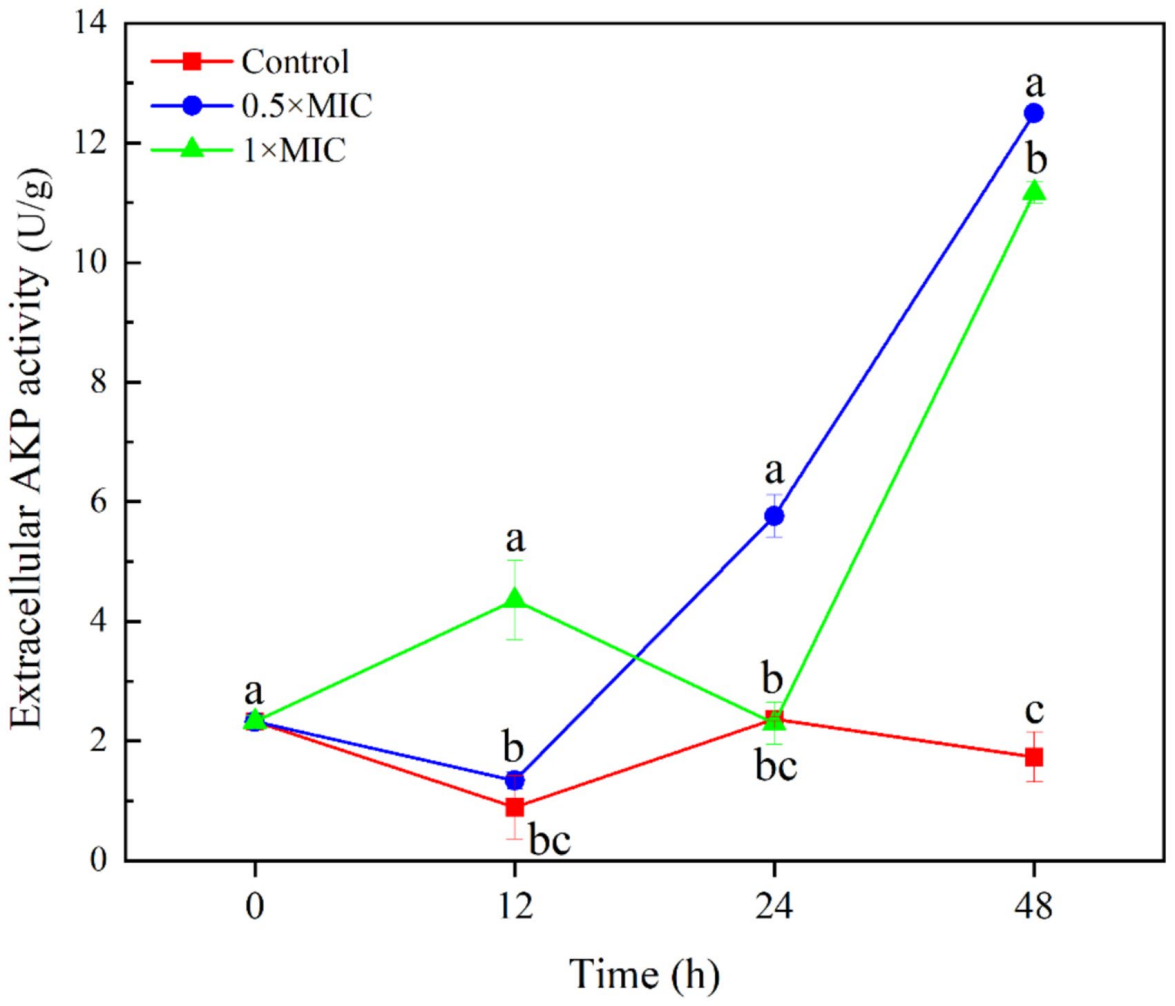
The inhibitory effect of curcumin-loaded nanoliposomes on *A. alstroemeriae* extracelluar AKP activity. Values are the mean ± standard deviation (SD) of three replicates. Different superscripts letters mean significantly different (*p* < 0.05).

The extracellular AKP activity of the 1/2MIC treatment group was 5.762 U/g, which significantly higher than that of the control group with value 2.365 U/g at 24 h (*p* < 0.05). The extracellular AKP activity of the 1/2MIC treatment group was 12.493 U/g, and the extracellular AKP activity of the 1MIC treatment group was 11.170 U/g, both significantly higher than the control group with value 1.733 U/g at 48 h (*p* < 0.05). This indicates that treatment with 1/2MIC and 1MIC curcumin-loaded nanoliposomes after 48 h, the cell wall of the *A. alstroemeriae* was damaged (Figure 3).

### 3.5 *In vivo* inhibitory effects of curcumin-loaded nanoliposomes against *Alternaria alstroemeriae*

#### 3.5.1 Inhibitory effects of curcumin-loaded nanoliposomes on *Alternaria alstroemeriae* growth in citrus fruit

**Figure 4:**
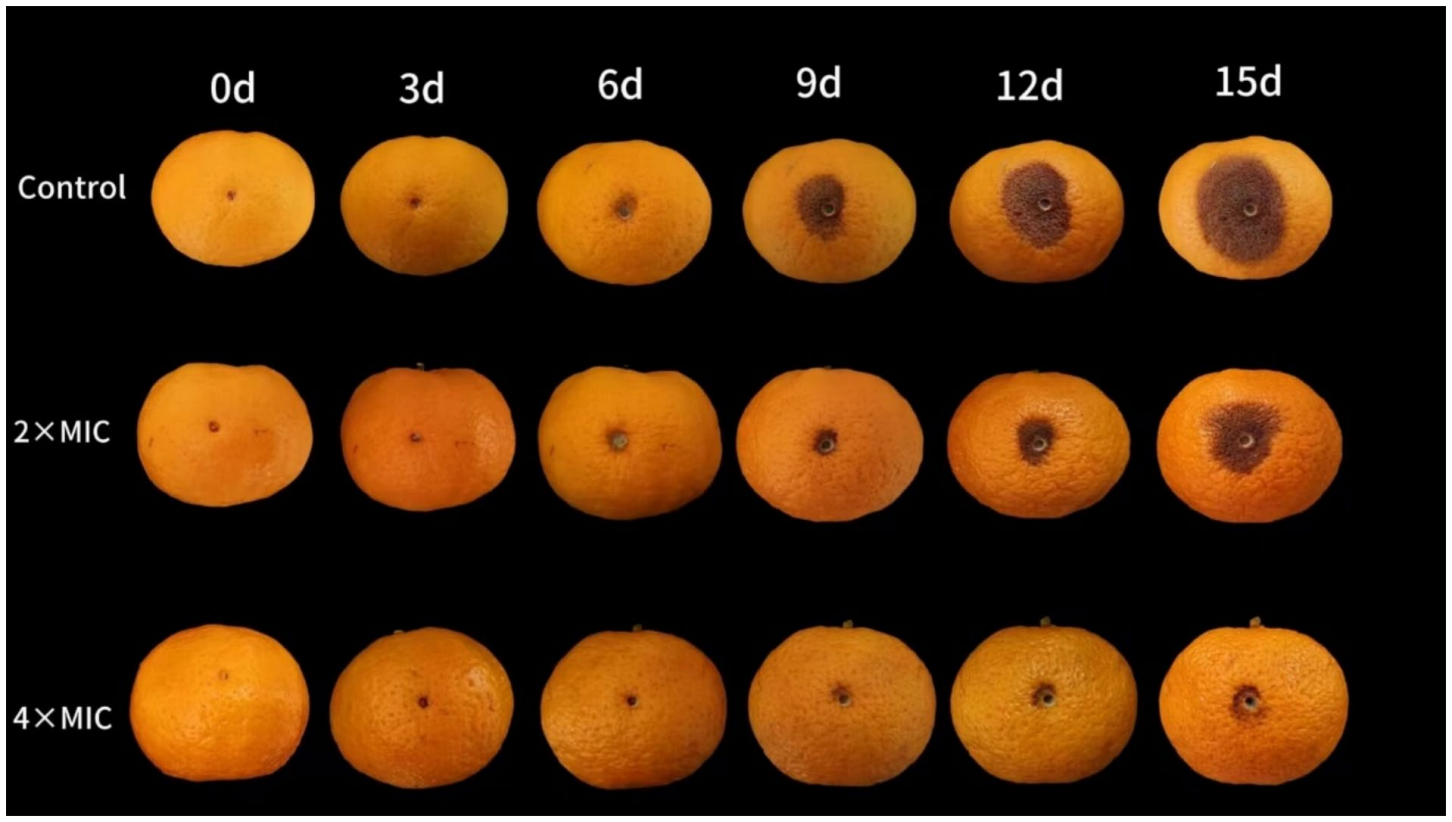
The inhibitory effect of curcumin-loaded nanoliposomes on *A. alstroemeriae* growth *in vivo.*

**Figure 5:**
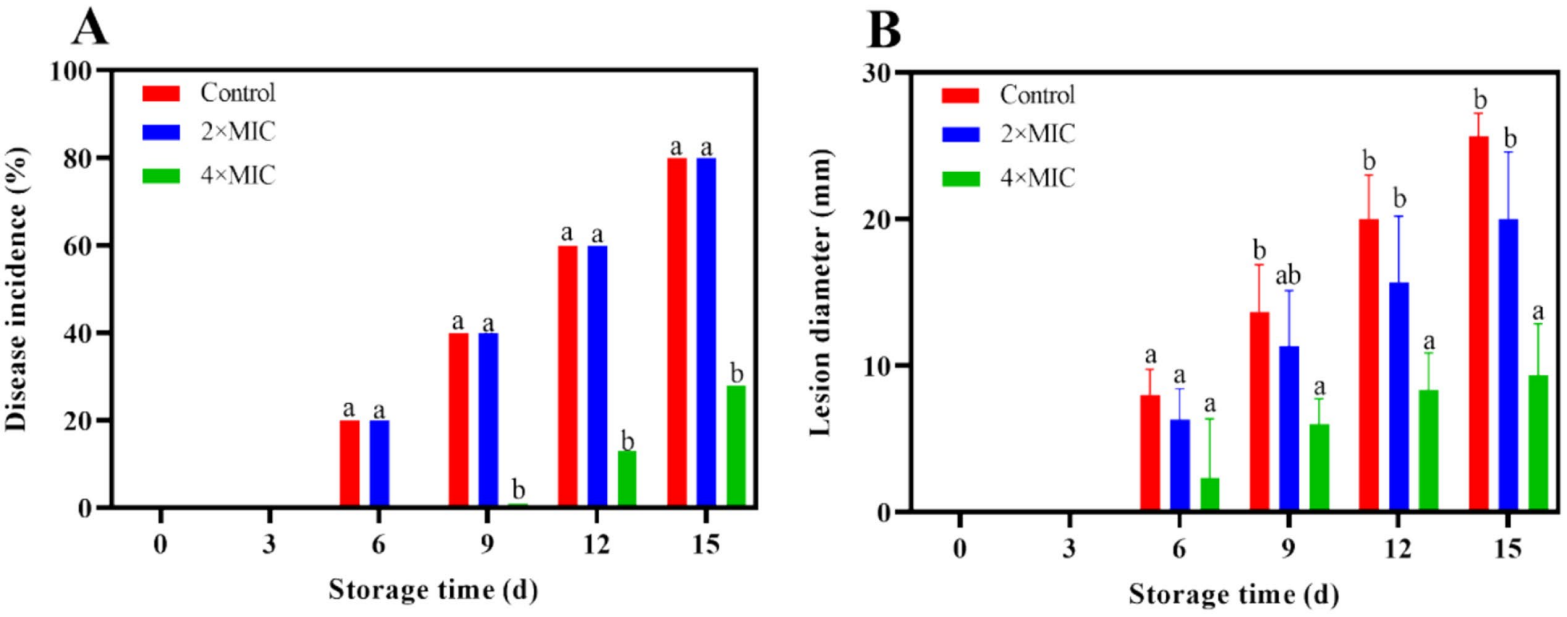
Effect of curcumin-loaded nanoliposomes on disease incidence **(A)** and lesion diameter **(B)** in *Citrus reticulata* Blanco ‘Orah’ peel inoculated with *A. alstroemeriae.* Values are the mean ± standard deviation (SD) of three replicates. Different superscripts letters mean significantly different (*p* < 0.05).

The citrus fruits of both the control group and the 2MIC treatment group showed decay at days 6. At this time, lesions appeared around the wound in both groups, and the texture of the flavedo at the wound site became softer. The fruit of the 4MIC treatment group showed symptoms of decay at the days 9 (Figure 4). The disease incidence of the 2MIC and 4MIC treatment group were significantly lower than that of the control group (*p* < 0.05; Figure 5A). As the storage time extension, the lesion of the fruits gradually expanded (Figure 5B). The treatment by 2MIC and 4MIC curcumin-loaded nanoliposomes can delay the occurrence of *A. alstroemeriae* on citrus fruit, and the treatment by 4MIC has the best effect. On the 15th day of storage, the lesion diameter of control, 2MIC and 4MIC treatment groups was 25.67 mm, 20 mm and 9.3 mm, the lesion diameter in the 2MIC and 4MIC treatment groups was 22.09 and 63.65% lower than that in the control group, respectively.

#### 3.5.2 Effects of curcumin-loaded nanoliposomes on TSS, titratable acidity and ascorbic acid contents of citrus fruit

**Figure 6:**
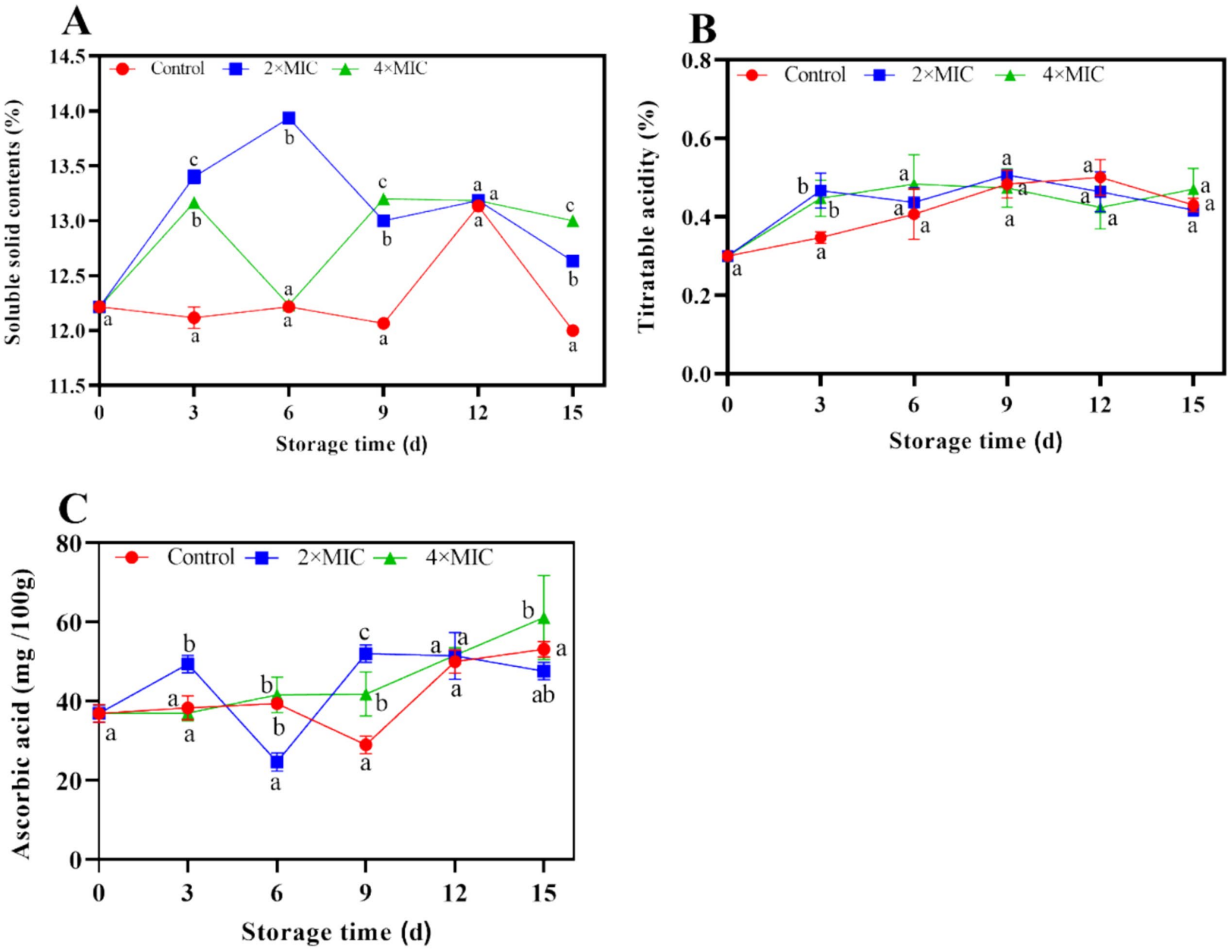
Effect of curcumin-loaded nanoliposomes on soluble solids **(A)**, titrateable acidity **(B)** and ascorbic acid **(C)** contents in *Citrus reticulata* Blanco ‘Orah’ peel inoculated with *A. alstroemeriae.* Values are the mean ± standard deviation (SD) of three replicates. Different superscripts letters mean significantly different (*p* < 0.05).

The TSS content of the control group was lower than that of the treatment group. The TSS content of the control group, 2MIC and 4MIC treatment groups were 12, 12.63 and 13%, respectively at days 15. With compared with the control group, the treatment group effectively maintained the TSS content of citrus fruit during storage, and the 4MIC treatment group had the best effect (Figure 6A). The TA content of the control group increased gradually, reaching its peak at 0.5% on days 12. The TA content of the liposome treated fruits showed a trend of first increasing and then decreasing during storage. On the days 15, the TA content of the control group, 2MIC group and 4MIC group was 0.43, 0.41, and 0.47%, respectively. There was no significant difference in the TA content change between the control group and the liposome treatment group (*p* > 0.05; Figure 6B).

During the storage period of days 9–15, the ascorbic acid content in the control group showed an upward trend, the 2 MIC treatment group showed a decreasing trend, the 4MIC group increased gradually. Compared with the control group and the 2MIC treatment group, the 4MIC curcumin liposome treatment significantly increased the ascorbic acid content of the fruit (*p* < 0.05). On the days 15, the ascorbic acid content of the 4MIC group increased by 14.98 and 28.38%, respectively, compared to the control group and the 2MIC group (Figure 6C).

#### 3.5.3 Effects of curcumin-loaded nanoliposomes on total phenolic and flavonoid contents of citrus fruit

**Figure 7:**
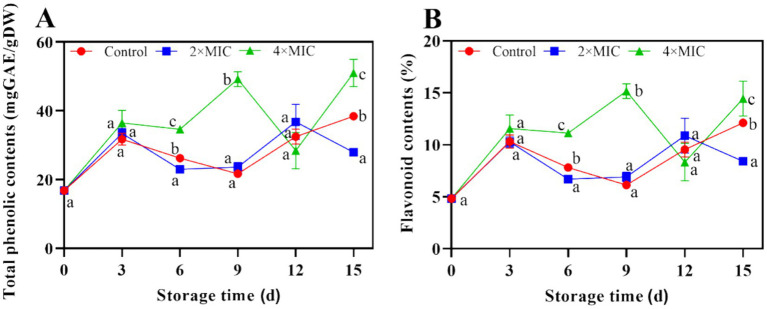
Effect of curcumin-loaded nanoliposomes on total phenolic **(A)** and flavoniod **(B)** contents in *Citrus reticulata Blanco* ‘Orah’ peel inoculated with *A. alstroemeriae.* Values are the mean ± standard deviation (SD) of three replicates. Different superscripts letters mean significantly different (*p* < 0.05).

There were significant differences in the total phenolic and flavonoid content between the control group and the 2MIC and 4MIC treatment groups (*p* < 0.05) at the days 15. The total phenolic and flavonoid contents of 2MIC treatment group were 27.34 and 3.71% lower than the control group, respectively. The total phenolic and flavonoid contents in the 4MIC curcumin treatment group were higher than those in the control group, with contents 32.75 and 2.33% higher, respectively. Overall, treatment with 4MIC effectively promoted an increase in total phenolic content and flavonoid substances (Figures 7A,B).

#### 3.5.4 Effects of curcumin-loaded nanoliposomes on the activity of antioxidant enzymes of citrus fruit

**Figure 8:**
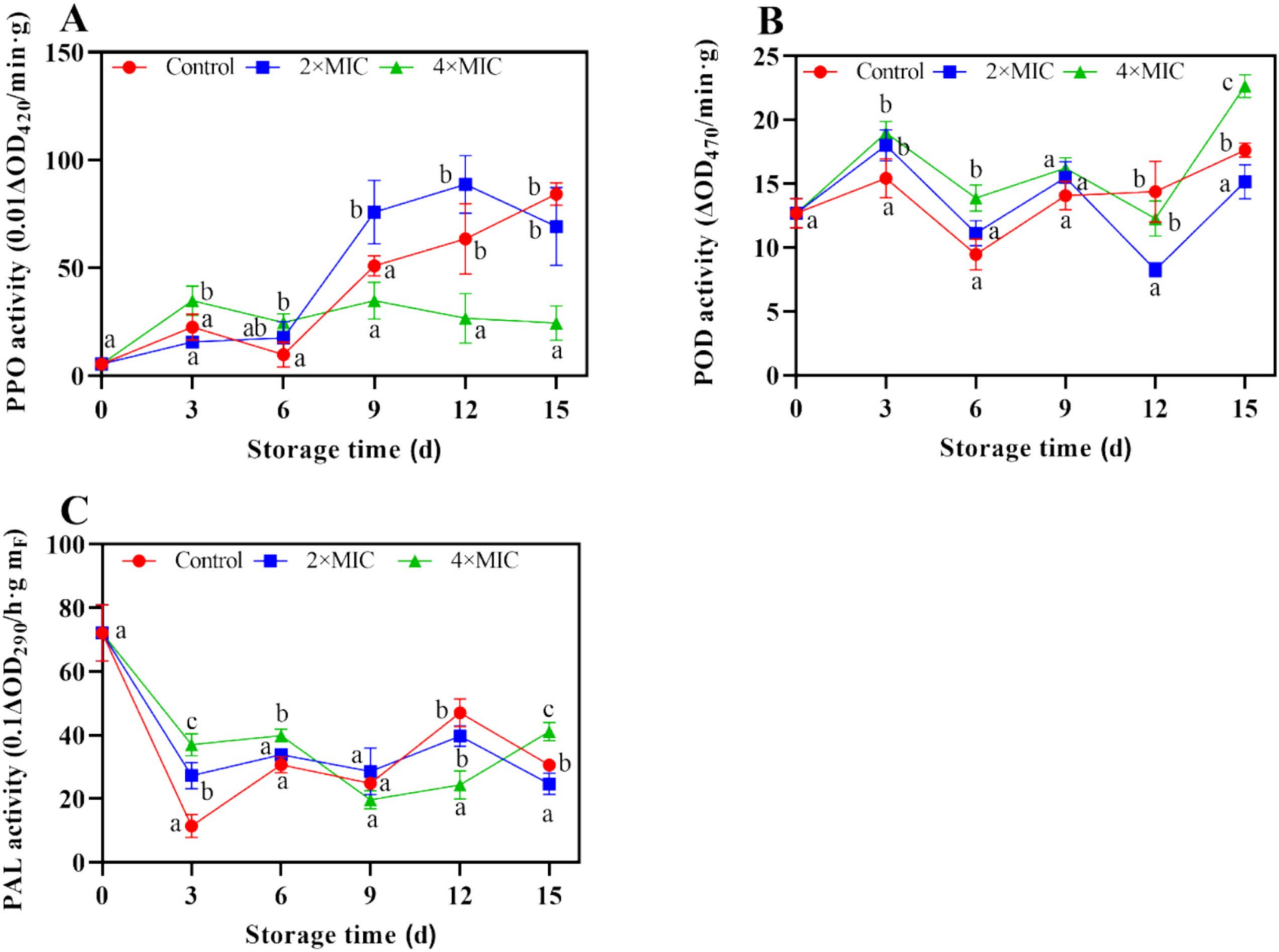
Effect of curcumin-loaded nanoliposomes on PPO **(A)**, POD **(B)** and PAL **(C)** activity in *Citrus reticulata* Blanco ‘Orah’ peel inoculated with *A. alstroemeriae.* Values are the mean ± standard deviation (SD) of three replicates. Different superscripts letters mean significantly different (*p* < 0.05).

As shown in Figure 8A, the treatment by 2MIC significantly enhanced the PPO activity, peaked at 88.63 U/g on days 12, and was 1.40-folds higher than that of control. Generally, curcumin-loaded nanoliposomes treatments resulted in increased activity of the antioxidant enzymes. The peroxidase (POD) activity continued to increase with the extension of storage, while curcumin-loaded nanoliposomes treatments induced higher POD activities during 0–3 d of storage. At the end of storage, the POD activities of 4MIC treated fruit were 1.29-fold than the control (Figure 8B). The phenylalanine ammonia lyase (PAL) activity in the control, 2MIC and 4MIC treatments showed fluctuation during storage. PAL activity in the 2MIC and 4MIC treatments were 2.38 and 3.22-fold than the control on day 3 (Figure 8C).

**Figure 9:**
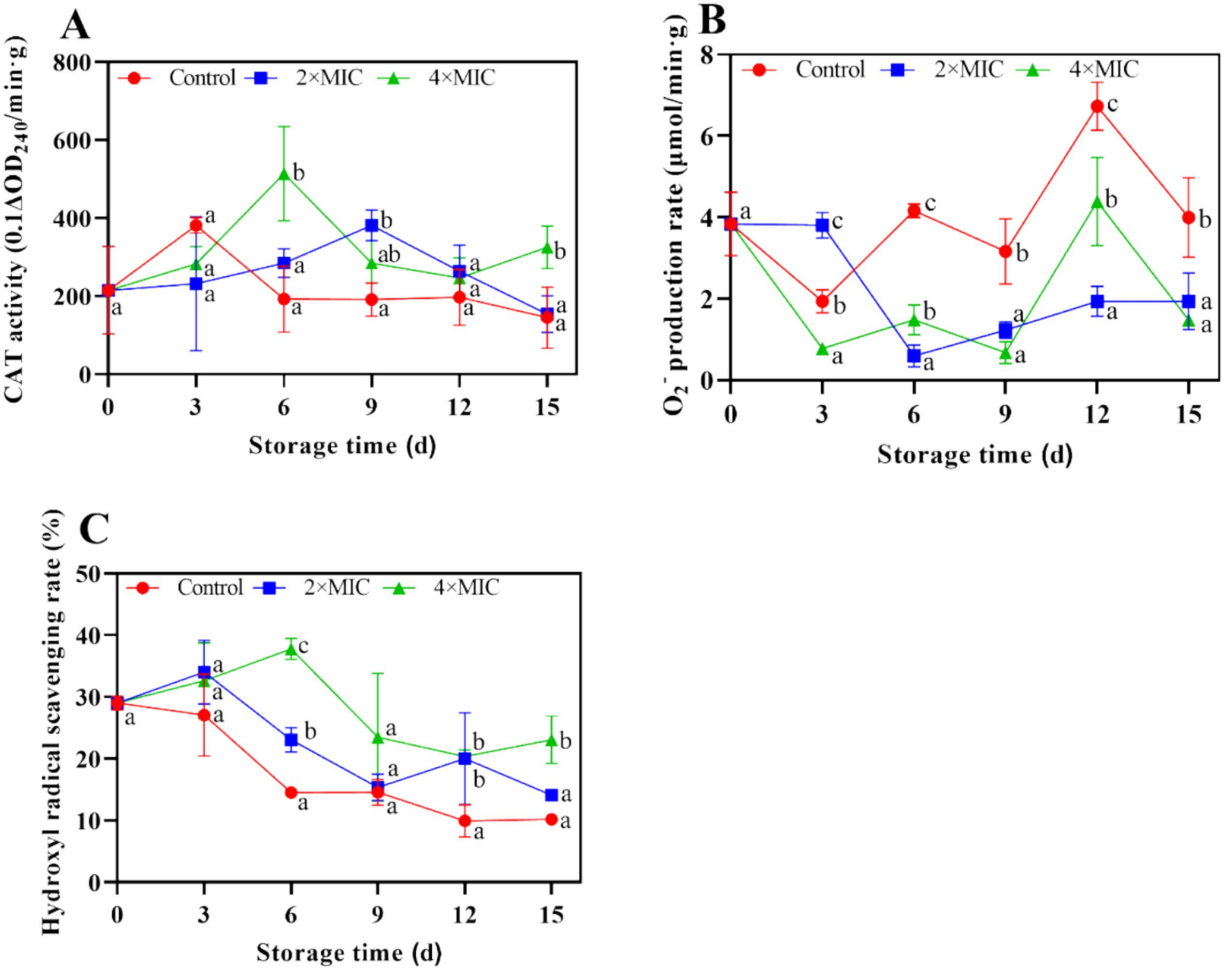
Effect of curcumin-loaded nanoliposomes on CAT activity **(A)**, O_2_^−^ production rate **(B)** and hydrogen radical scavenging rate **(C)** activity in *Citrus reticulata* Blanco ‘Orah’ peel inoculated with *A. alstroemeriae.* Values are the mean ± standard deviation (SD) of three replicates. Different superscripts letters mean significantly different (*p* < 0.05).

The changes in catalase (CAT) activity in the control group were the same as those in treatment group, which showed a trend of rapid increase first and decline afterwards. The CAT activity of the treatment group was significantly higher than the control group on days 15 (Figure 9A). The O_2_^−^ production rate in citrus fruit were shown in Figure 9B, respectively. There was a sharp decrease in the production of O_2_^−^ within the initial 3 days. The O_2_^−^ production remained at the higher level of control in comparison with treatment on day 12. According to Figure 9C, the ·OH radical scavenging activity of control was lower than the treatment group between day 0 and 15, the difference between the treated fruit and control was significant during storage.

**Figure 10:**
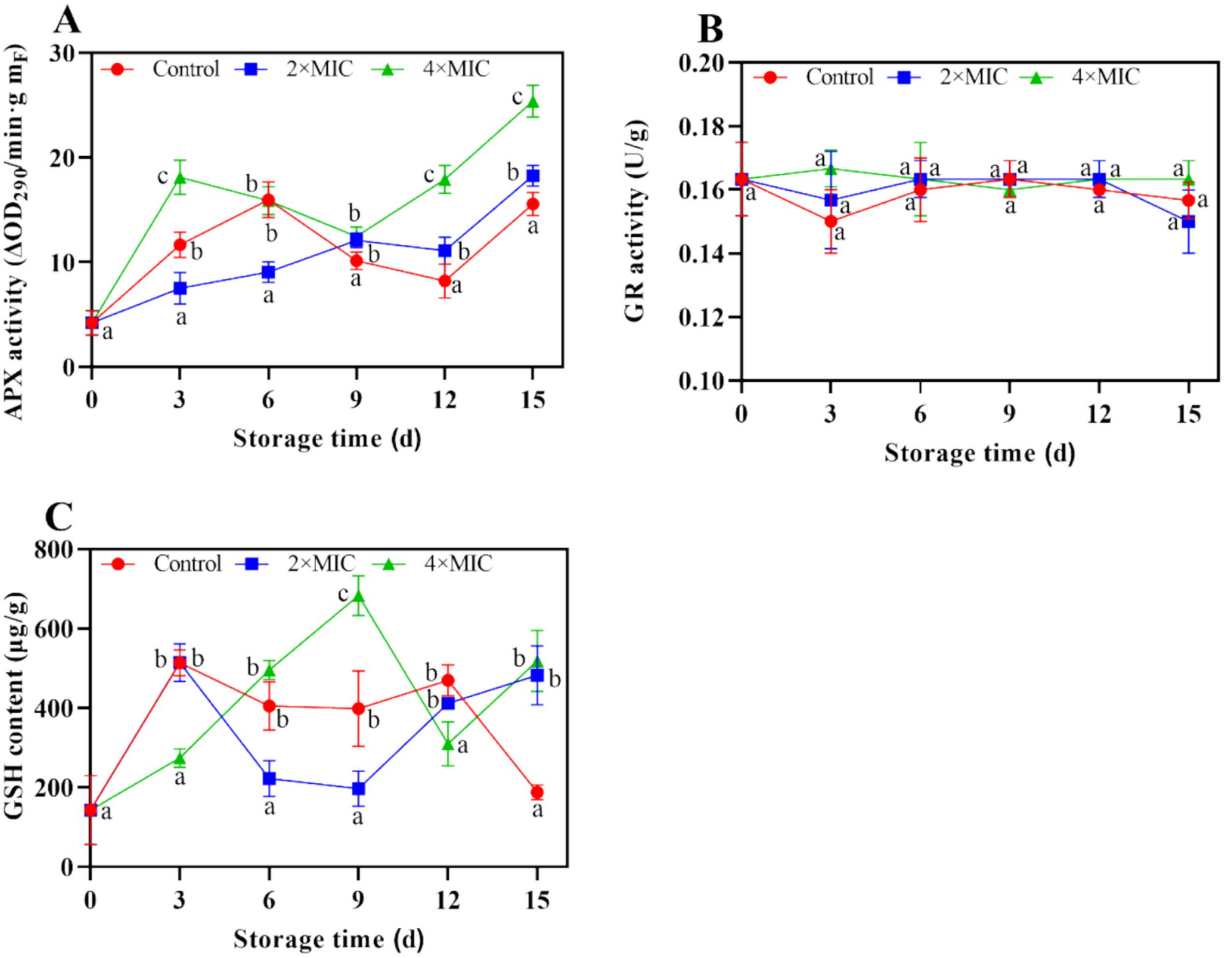
Effect of curcumin-loaded nanoliposomes on APX activity **(A)**, GR activity **(B)** and GSH content **(C)** in *Citrus reticulata* Blanco ‘Orah’ peel inoculated with *A. alstroemeriae.* Values are the mean ± standard deviation (SD) of three replicates. Different superscripts letters mean significantly different (*p* < 0.05).

The ascorbate peroxidase (APX) activity of the treatment group and the control group gradually increased. The APX activity of the control group was lower than that of the treatment group during day 9 and day 15, respectively (Figure 10A). As illustrated in Figure 10B, the glutathione reductase (GR) activity of the treatment group showed a fluctuation trend throughout the storage period, while the GR activity of the 4MIC treatment group increased in the 0–3 days of storage but then started to decrease. The reduced glutathione (GSH) content of the 4MIC treatment group showed increased sharply from day 0 to day 9, which significantly higher than the 2MIC and control group. While the GSH content of the CK group increased in the 0–3 days of storage but then started to decrease (Figure 10C).

## 4 Discussions

*Alternaria* is composed of species that may be saprophytic, endophytic, or pathogenic in nature (Meena et al., 2017). Black rot caused by *Alternaria* is one of the most important diseases of citrus, which can reduce crop yield and cause considerable economic losses to farmers and food industries (Cuenca et al., 2016), The production of host specific toxins can lead to necrotic lesions in citrus fruits and young leaves, as well as fruit drop in susceptible genotypes (Felipini et al., 2023). This study isolated *A. alstroemeriae* from naturally decayed citrus fruits after harvesting and identified it through morphological characteristics and rDNA-ITS analysis. The isolated strains were subjected to sequence alignment and phylogenetic tree analysis, and the results showed a high match with the previously reported *A. alstroemeriae* from citrus fruit (Cara et al., 2022).

The antifungal experiments *in vitro* indicated that curcumin-loaded nanoliposomes can significantly inhibit the growth of *A. alstroemeriae* in a dose-dependent manner. *In vivo* inoculation experiments showed that curcumin-loaded nanoliposomes significantly inhibited the occurrence of citrus black rot disease without any adverse effects on fruit quality. Curcumin-loaded nanoliposomes reduced the incidence of postharvest diseases in citrus fruits, indicating that curcumin-loaded nanoliposomes maintain the good inhibitory properties of curcumin and overcome its water solubility issues. They may have good application prospects in controlling postharvest diseases in citrus fruit. Similar results have been reported previously that the curcumin-loaded electrospun zein nanofibers displayed great potential in controlling *Botrytis cinerea* and *Penicillium expansum* of apples (Yilmaz et al., 2016). The above results indicated that inclusion complexes are an effective method to overcome the practical application problems of curcumin.

Previous studies have shown that some substances exhibited their antifungal activity by disrupting the permeability of cell walls and membrane integrity of pathogen (Shu et al., 2024; Zhao et al., 2024). AKP is an intracellular enzyme formed in the cytoplasm, which leaks from the cytoplasm into the extracellular environment when the permeability of the fungi cell wall was impaired (Zhang et al., 2024). In our study, the extracellular AKP activity of *A. alstroemeriae* increased after curcumin-loaded nanoliposomes treatment, indicating that the cell wall may be the antifungal target of curcumin-loaded nanoliposomes. Similar studies indicated that black pepper essential oil played an antifungal role by changing the cell structure and destroying the cell wall, and black pepper essential oil can lead to the leakage of AKP in *A. flavus* (Zhang et al., 2021).

In this study, the treatment by 10 μmol/L curcumin-loaded nanoliposomes effectively inhibited the infection of *A. alstroemeriae* on citrus fruits. In addition, the fruit has resistance reactions when infected by pathogens, the enzymes of SOD, CAT, POD and APX play a crucial role in scavenging ROS (Lei et al., 2024). The enzyme CAT and POD can degrade free radicals and H_2_O_2_, maintaining the balance of intracellular ROS, and protect the structural stability of the plasma membrane (Wang et al., 2023). In our research, the enhanced activities of antioxidant enzymes CAT, POD and APX were observed in curcumin-loaded nanoliposomes treated citrus fruit. Ding et al. (2024) found that acid electrolytic water treatment significantly enhanced the activity and expression of beneficial antioxidant-related enzymes such as superoxide dismutase (SOD), catalase (CAT), ascorbate peroxidase (APX), aiding in ROS clearance and reducing membrane lipid peroxidation. Furthermore, melatonin treated broccoli suppress the over accumulation of superoxide anion O_2_^−^ and hydrogen peroxide H_2_O_2_ (Yan et al., 2024). In our study, the O_2_^−^ production rate of citrus fruits treated with curcumin-loaded nanoliposomes was significantly lower than that of control fruits, indicating that curcumin liposome treated fruits can reduce the concentration of intracellular H_2_O_2_ and MDA, which is consistent with previous research results.

The phenylalanine ammonia lyase (PAL) and polyphenol oxidase (PPO) in the phenylpropanoid metabolic pathway play a crucial role in fruit disease resistance, mainly involved in the biosynthesis of secondary metabolites such as flavonoids, phenols, and lignin, which closely related to plant disease resistance (Duan et al., 2021). The results showed that the content of total phenolic and flavonoid compounds in citrus fruits increased after treatment with curcumin-loaded nanoliposomes, while also enhancing the activity of PAL and PPO, thereby increasing the disease resistance of the citrus fruit. ASA-GSH cycle plays a crucial role in plant ROS metabolism (Zhou et al., 2024). The ascorbic acid is an efficient antioxidant that can eliminate the adverse effects of excessive H_2_O_2_ in plants (Yao et al., 2021). Li et al. (2024) also found that starch-based films reinforced with curcumin-loaded nanocomplexes treatment induced the accumulation of ASA in blueberries. It is noteworthy that the ascorbic acid levels in the curcumin-loaded nanoliposomes treatment were always greater than those in the control group, especially in the middle and late storage periods. These results were also confirmed by the accumulation of ascorbic acid and GSH in the curcumin liposome treatment group reduced oxidative damage caused by postharvest disease infection in citrus fruits. Liu et al. (2021) also concluded that the application of exogenous ascorbic acid was able to significantly detoxify ROS as well as MDA biosynthesis by triggering the operation of the antioxidant system in longan fruit. Huang et al. (2022) reported that acibenzolar-S-methyl could delay pears flesh firmness declined by suppressing ROS metabolites accumulation by increasing AsA and GSH contents and GR activity.

In our study, the curcumin-loaded nanoliposomes significantly increased the content of total phenols and flavonoids in citrus, and promoted their maximum accumulation on the 9th day. Increased contents of phenols and flavonoids would thus limit growth of *A. alstroemeriae* hence slowing down spread of black rot disease. Similar results have previously been reported by Kong et al. (2024), suggesting that production of total phenols effectively conferred disease resistance to *Aspergillus carbonarius* in grape. The total soluble solids, titratable acid and ascorbic acid contents of fruits are important indicators for evaluating their sensory and nutritional quality. In this study, compared with the control fruit, the curcumin-loaded nanoliposomes treated fruit showed a significantly stable quality during the storage period, demonstrating the potential of curcumin-loaded nanoliposomes for maintaining postharvest citrus fruit quality and flavor.

## 5 Conclusion

In this study, we successfully isolated and identified *A. alstroemeriae* from local citrus fruit. We found that curcumin-loaded nanoliposomes could significantly inhibit mycelia growth of *A. alstroemeriae*. The increase in AKP activity is the evidence that curcumin-loaded nanoliposomes disrupt the integrity of the cell wall of pathogen. The curcumin-loaded nanoliposomes increased the content of total phenol, flavonoids, ascorbic acid and GSH, activated antioxidant enzymes POD, PPO, CAT, GR and APX in citrus fruit to remove excess reactive oxygen species and prevent plant cell damage. Curcumin-loaded nanoliposomes treatment maintained the quality of citrus fruit by delaying the TSS, TA degradation and higher level of total phenolics and flavonoid contents in citrus fruit. Further studies are recommended to investigate the resistance mechanism through transcriptome and metabolome analysis to find genes and metabolites are related to disease-resistant in citrus fruit. Our findings laid the theoretical groundwork for future applications in the postharvest disease control of citrus. Curcumin-loaded nanoliposomes have antifungal effect against citrus postharvest diseases, it can be used to as a prospective biological preservative in citrus industry.

## Data Availability

The original contributions presented in the study are included in the article/Supplementary material, further inquiries can be directed to the corresponding author/s.

## Supplementary material

SUPPLEMENTARY FIGURE S1 Front and reverse colony morphology of the isolated strains.

SUPPLEMENTARY FIGURE S2 Spore morphology of the isolated strains.

SUPPLEMENTARY FIGURE S3 **(A)** Decayed citrus fruits caused by *Alternaria alstroemeriae*. **(B)** Decayed citrus fruits caused by *Rhizopus arrhizus* **(C)** Decayed citrus fruits caused by *Aspergillus flavus* **(D)** Decayed citrus fruits caused by *Penicillium digitatum*.

SUPPLEMENTARY FIGURE S4 Phylogenetic tree of four isolated strains based on rDNA-ITS sequences. **(A)** *Alternaria alstroemeriae*. **(B)** *Rhizopus arrhizus*. **(C)** *Aspergillus flavus* **(D)** *Penicillium digitatum*.
