# Supplementary figures and images for: Isolation and identification of Alternaria alstroemeriae causing postharvest black rot in citrus and its control using curcumin-loaded nanoliposomes
Source: Front Microbiol. 2025 Mar 4;16:1555774. doi: 10.3389/fmicb.2025.1555774 (PMC11914099; doi:10.3389/fmicb.2025.1555774)

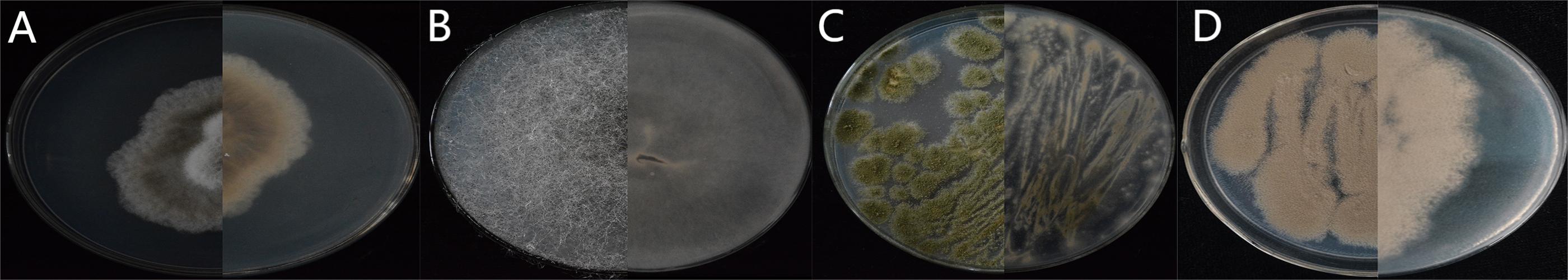

Supplement: SUPPLEMENTARY FIGURE S1 — Front and reverse colony morphology of the isolated strains. [file Image_1.JPEG]

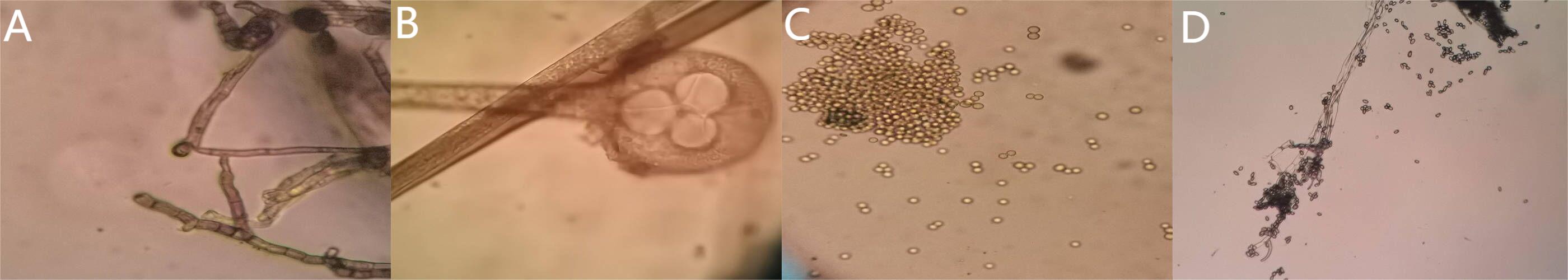

Supplement: SUPPLEMENTARY FIGURE S2 — Spore morphology of the isolated strains. [file Image_2.JPEG]

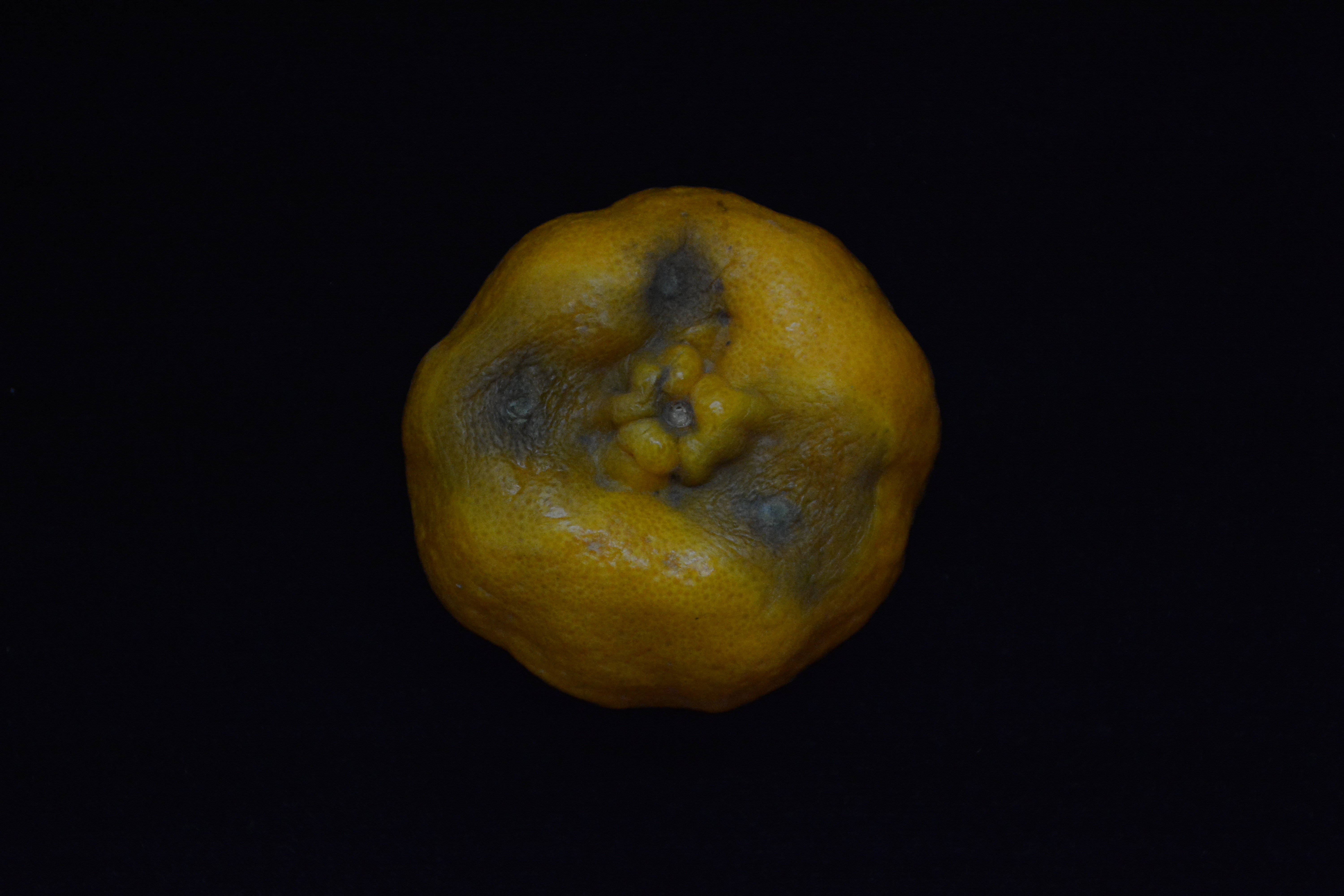

Supplement: SUPPLEMENTARY FIGURE S3 — (A) Decayed citrus fruits caused by Alternaria alstroemeriae. (B) Decayed citrus fruits caused by Rhizopus arrhizus (C) Decayed citrus fruits caused by Aspergillus flavus (D) Decayed citrus fruits caused by Penicillium digitatum. [file Image_3.JPEG]

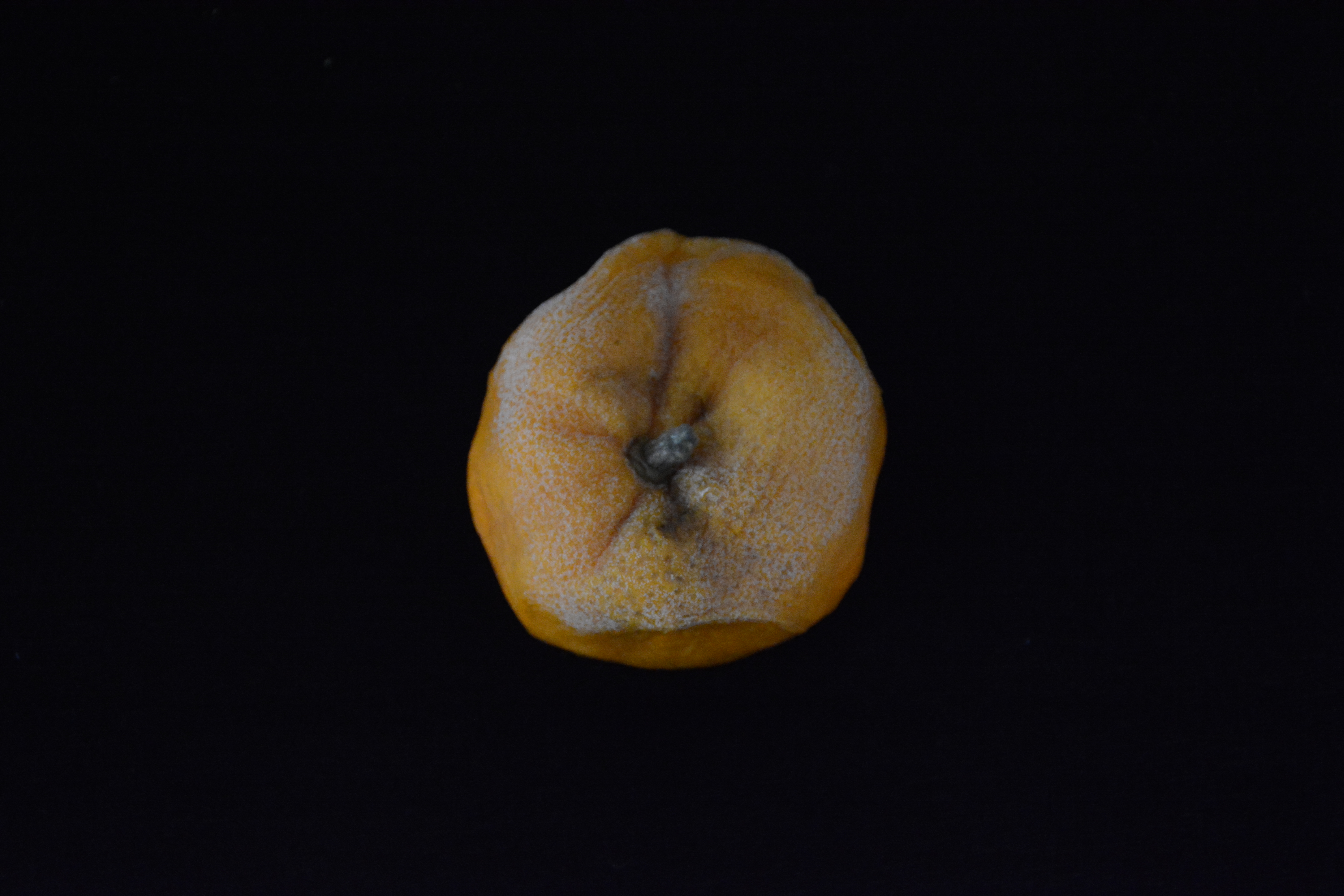

Supplement: SUPPLEMENTARY FIGURE S4 — Phylogenetic tree of four isolated strains based on rDNA-ITS sequences. (A) Alternaria alstroemeriae. (B) Rhizopus arrhizus. (C) Aspergillus flavus (D) Penicillium digitatum. [file Image_4.JPEG]

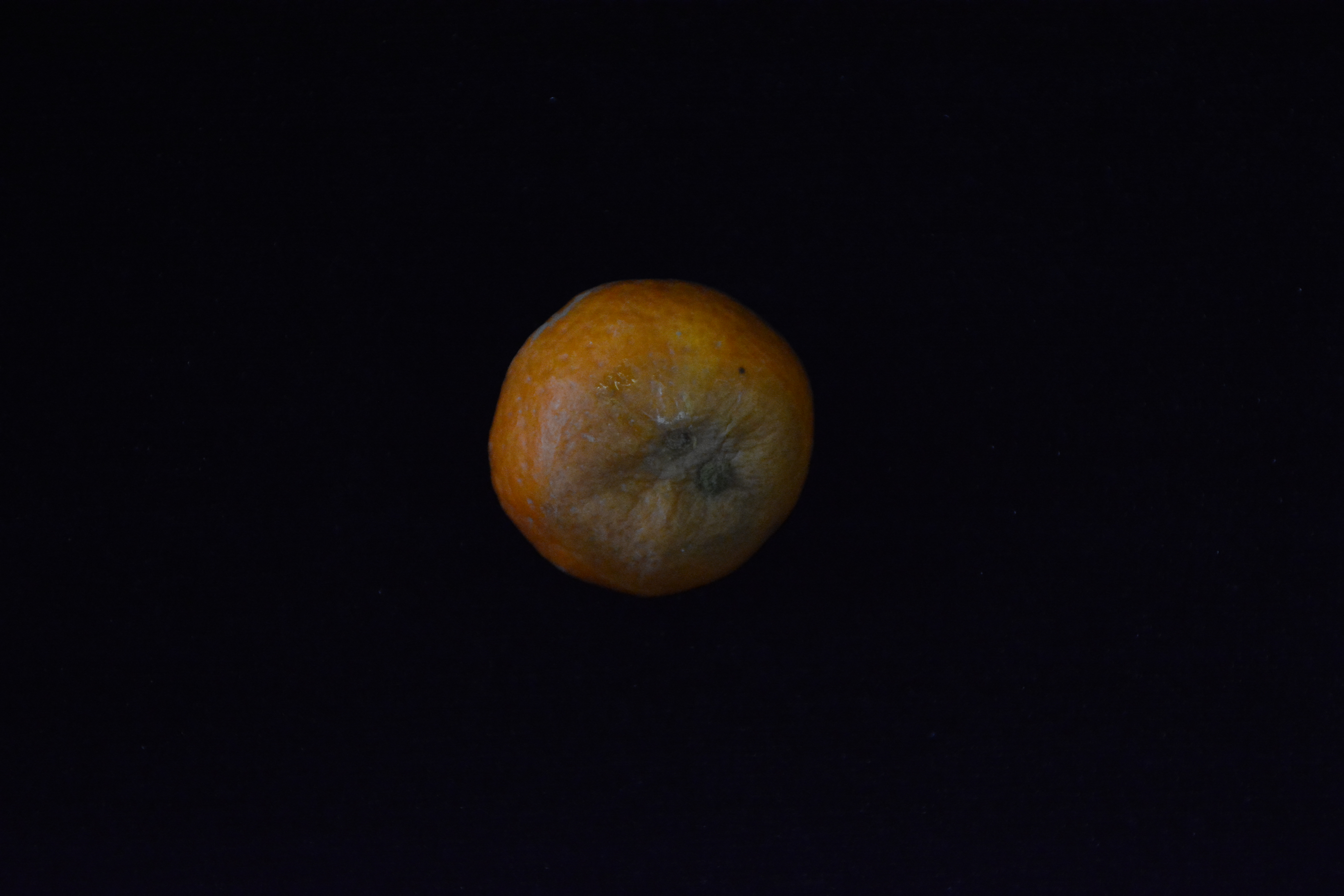

Supplement: Supplementary file 5 [file Image_5.JPEG]

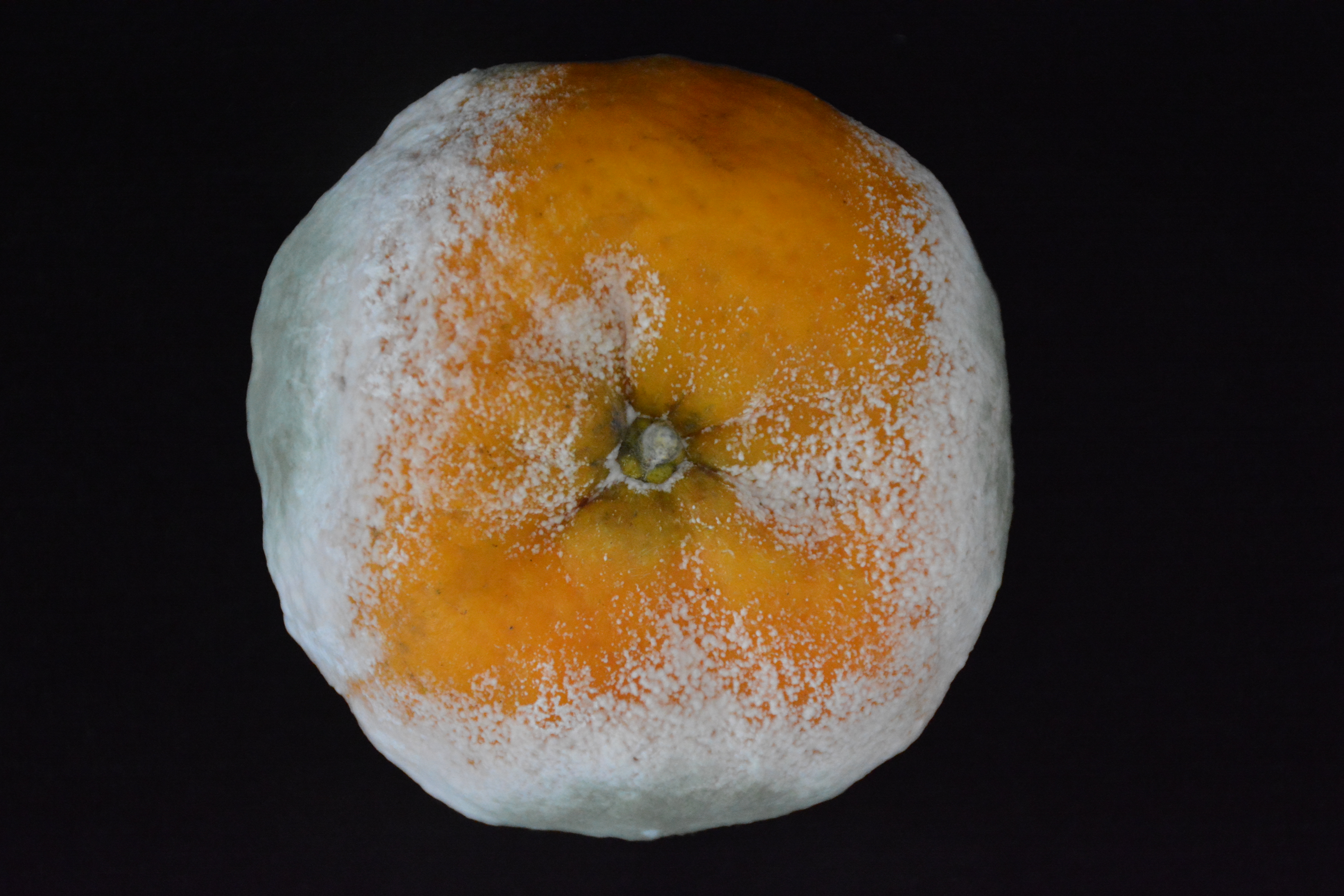

Supplement: Supplementary file 6 [file Image_6.JPEG]

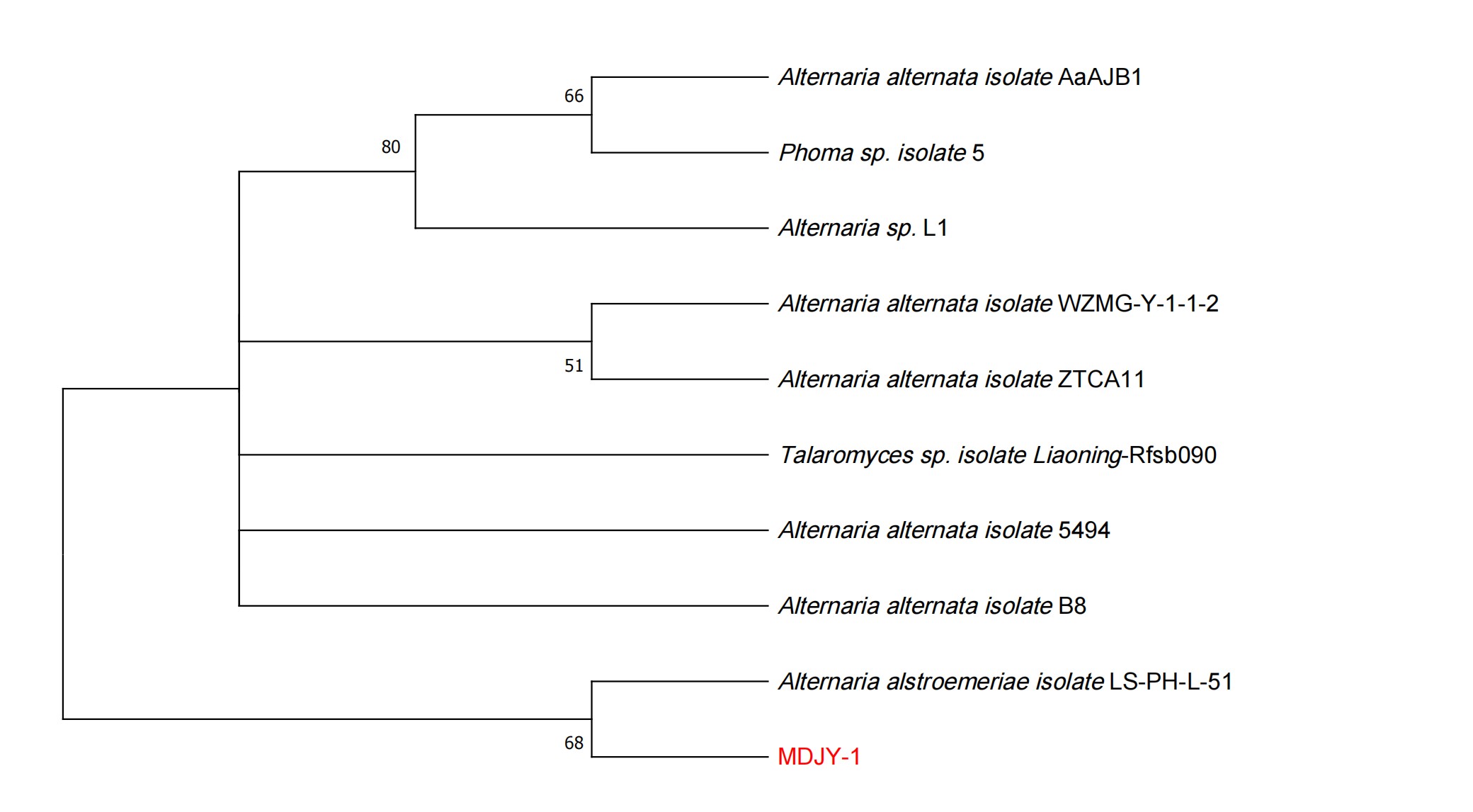

Supplement: Supplementary file 7 [file Image_7.JPEG]

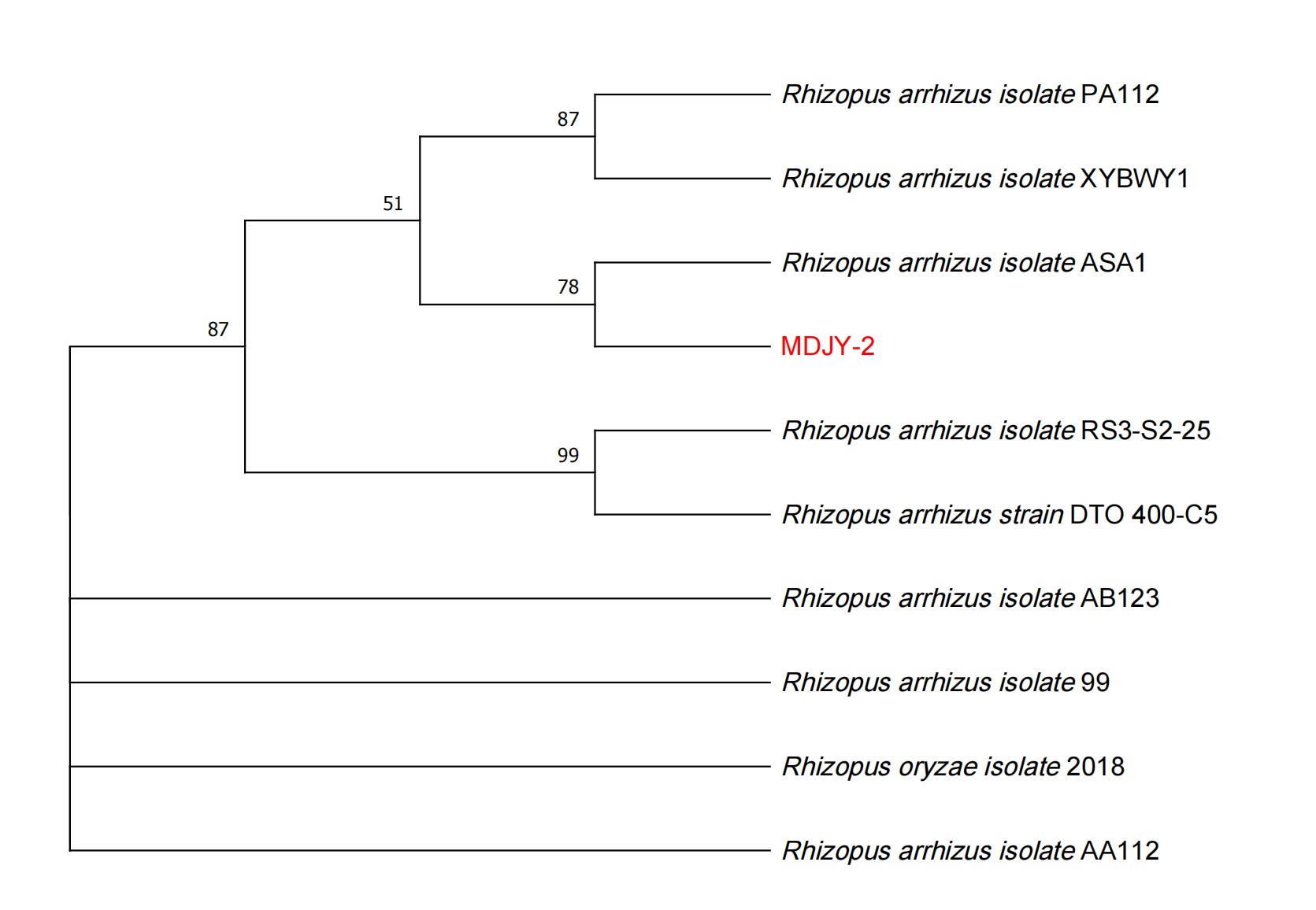

Supplement: Supplementary file 8 [file Image_8.JPEG]

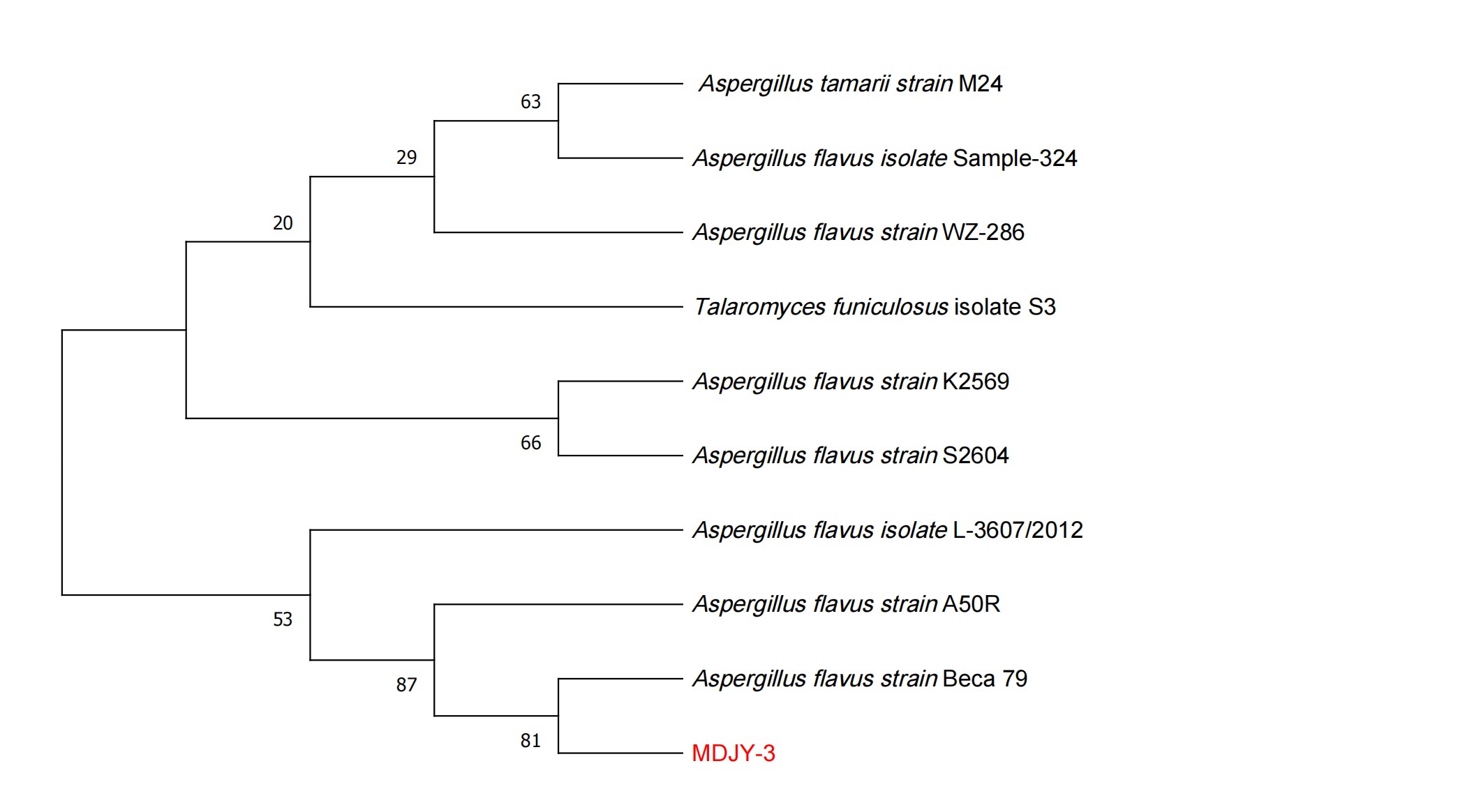

Supplement: Supplementary file 9 [file Image_9.JPEG]

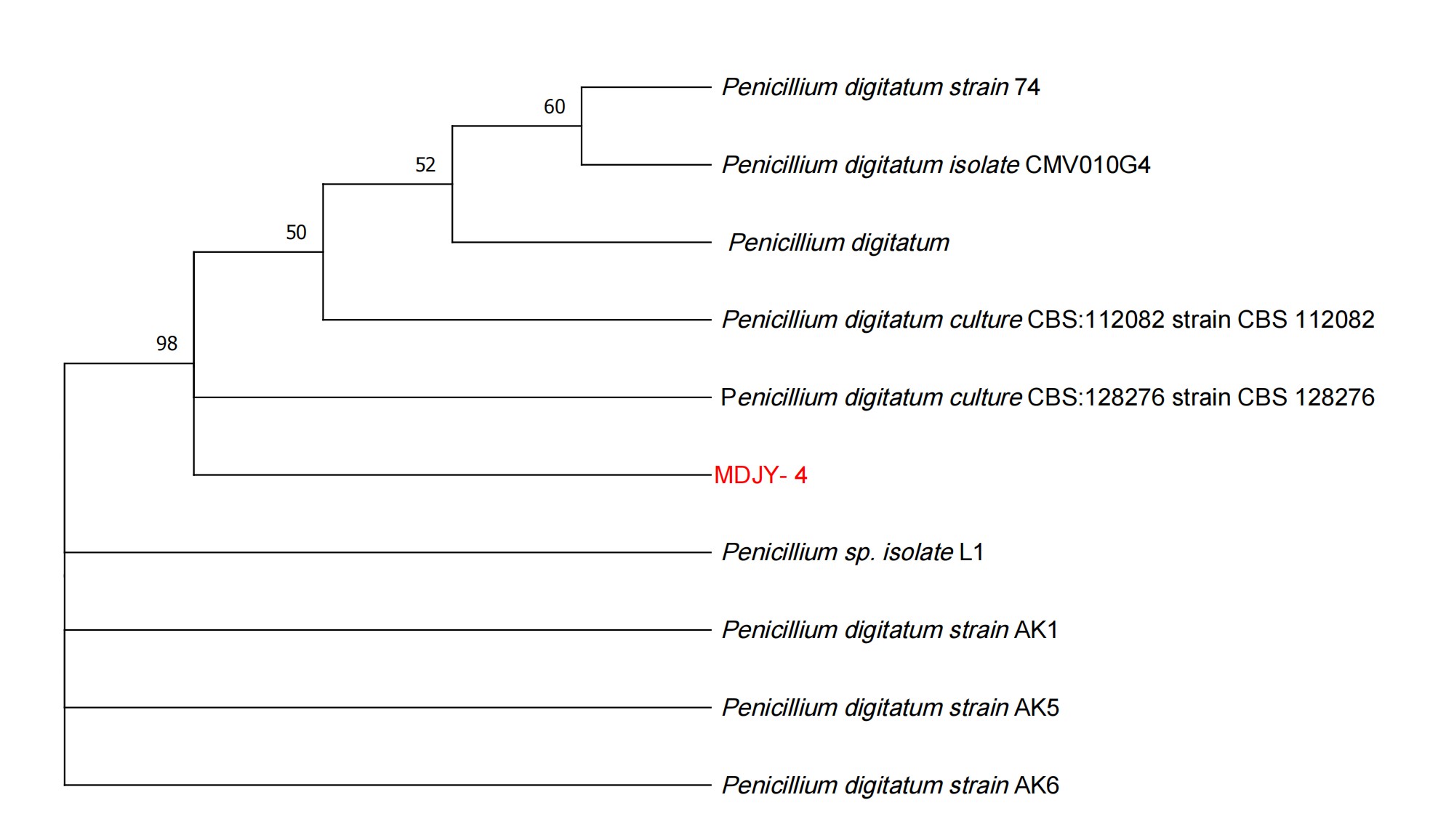

Supplement: Supplementary file 10 [file Image_10.JPEG]
